# Supplementary material for: Meta-analysis of 22,710 human microbiome metagenomes defines an oral-to-gut microbial enrichment score and associations with host health and disease
Source: Nat Commun. 2025 Dec 23;17:196. doi: 10.1038/s41467-025-66888-1 (PMC12780261; doi:10.1038/s41467-025-66888-1)
Supplement: Supplementary file 1 — Supplementary Information [file 41467_2025_66888_MOESM1_ESM.pdf]

Supplementary figures

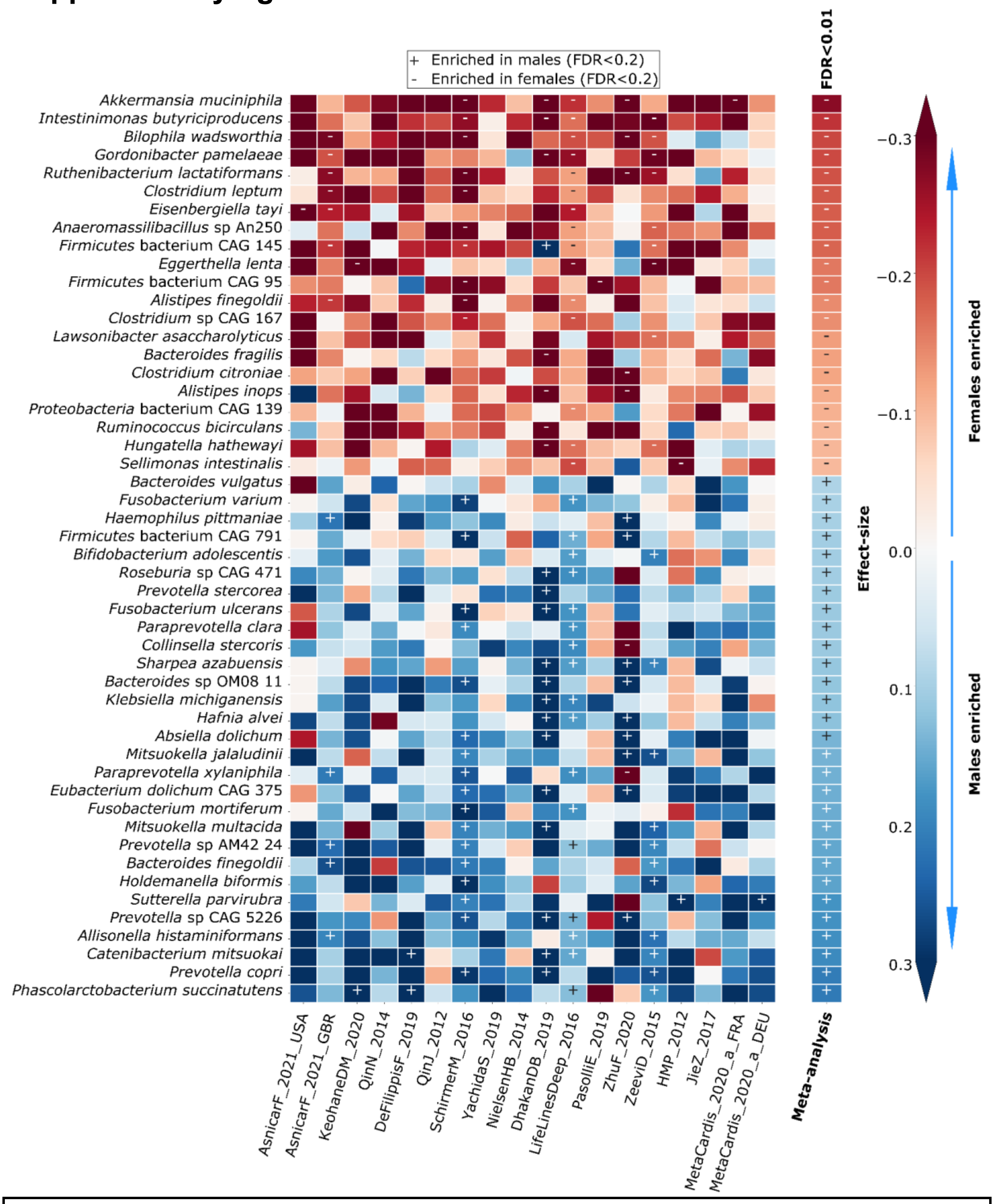

Supplementary Figure 1. Meta-analysis of 5,505 individuals' metagenomes from 18 datasets (3,288 females, 2,216 males) shows variations in the composition of the sex-associated microbiome that are not easily detected from the analysis of individual datasets. The 50 microbial species with the highest Standardized Mean Differences meta-analysis coefficient (Q<0.01) of male vs. female samples (computed as Standardized Mean Differences from a linear model controlling for age, BMI, and sequencing depth on centered log-ratio transformed species relative abundances) are reported. Red-blue heatmaps highlight the direction of the effect-size (blue: male, red: female). Signs mark the FDR significance (Q < 0.2 for single datasets, Q < 0.01 for the meta-analysis). Plus is used for the positive effect-sizes; minus is used for the negative effect-sizes.

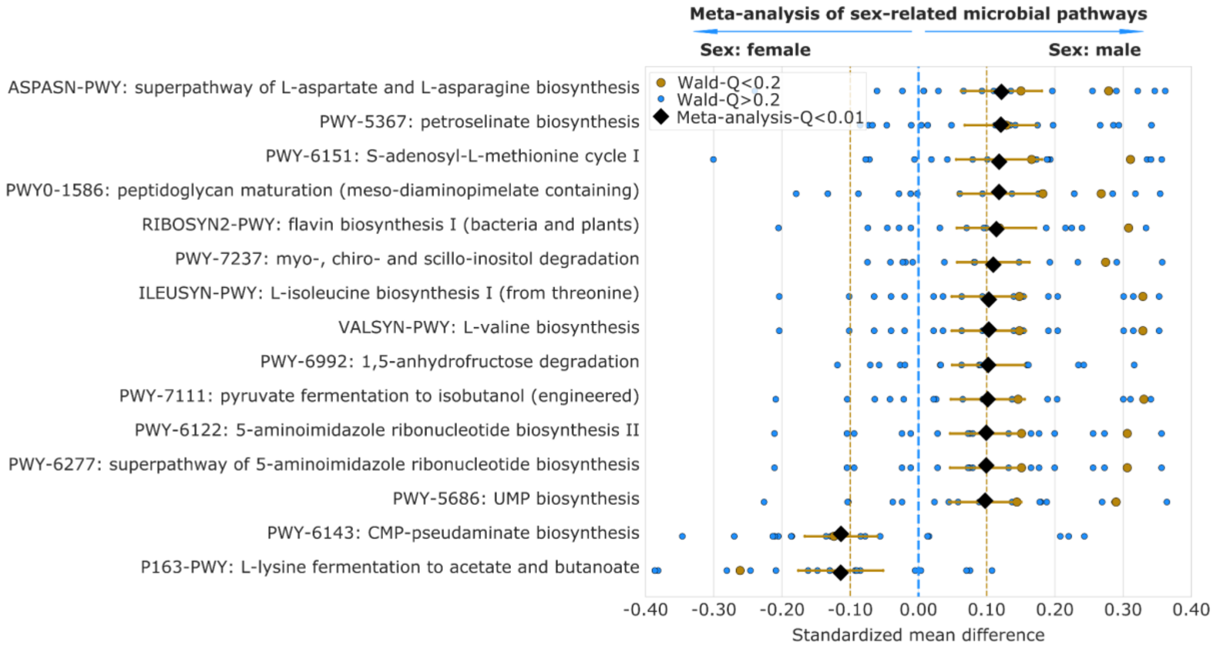

Supplementary Figure 2. Meta-analysis of 5,505 individual metagenomes from 18 datasets (3,288 females, 2,216 males) reveals sex-associated microbial differences in the Metacyc pathway potential of the healthy, adult, stool microbiome. The 30 microbial species and genera with highest Standardized Mean Differences meta-analysis coefficient (Q < 0.01) of male vs. female samples. Effect-sizes are computed as Standardized Mean Differences from a linear model controlling for age, BMI, and sequencing depth on centered log-ratio transformed species relative abundances. Yellow: significant effect-size (Q < 0.2). Light-blue: non significant effect-size. Black diamonds: Standardized Mean Differences between males and females synthesized by a meta-analysis. Yellow horizontal lines show 95% confidence intervals of the meta-analysis effect size.

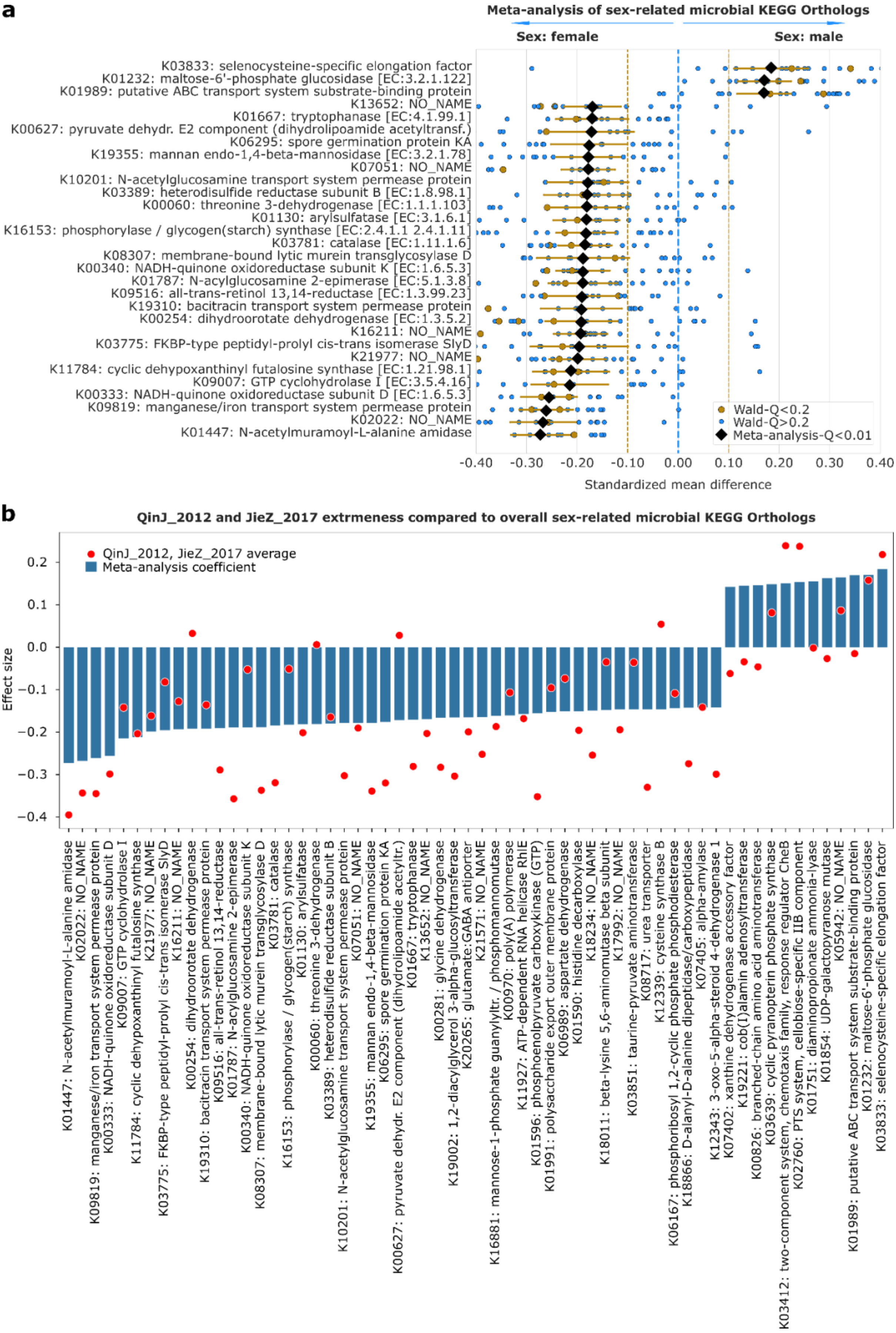

a
Meta-analysis of sex-related microbial KEGG Orthologs
Sex: female
Sex: male
K03833: selenocysteine-specific elongation factor
K01232: maltose-6'-phosphate glucosidase [EC:3.2.1.122]
K01989: putative ABC transport system substrate-binding protein
K13652: NO_NAME
K01667: tryptophanase [EC:4.1.99.1]
K00627: pyruvate dehydr. E2 component (dihydrolipoamide acetyltransf.)
K06295: spore germination protein KA
K19355: mannan endo-1,4-beta-mannosidase [EC:3.2.1.78]
K07051: NO_NAME
K10201: N-acetylglucosamine transport system permease protein
K03389: heterodisulfide reductase subunit B [EC:1.8.98.1]
K00060: threonine 3-dehydrogenase [EC:1.1.1.103]
K01130: arylsulfatase [EC:3.1.6.1]
K16153: phosphorylase / glycogen(starch) synthase [EC:2.4.1.1 2.4.1.11]
K03781: catalase [EC:1.11.1.6]
K08307: membrane-bound lytic murein transglycosylase D
K00340: NADH-quinone oxidoreductase subunit K [EC:1.6.5.3]
K01787: N-acylglucosamine 2-epimerase [EC:5.1.3.8]
K09516: all-trans-retinol 13,14-reductase [EC:1.3.99.23]
K19310: bacitracin transport system permease protein
K00254: dihydroorotate dehydrogenase [EC:1.3.5.2]
K16211: NO_NAME
K03775: FKBP-type peptidyl-prolyl cis-trans isomerase SlyD
K21977: NO_NAME
K11784: cyclic dehypoxanthinyl futalosine synthase [EC:1.21.98.1]
K09007: GTP cyclohydrolase I [EC:3.5.4.16]
K00333: NADH-quinone oxidoreductase subunit D [EC:1.6.5.3]
K09819: manganese/iron transport system permease protein
K02022: NO_NAME
K01447: N-acetylmuramoyl-L-alanine amidase
Wald-Q<0.2
Wald-Q>0.2
Meta-analysis-Q<0.01
-0.40 -0.30 -0.20 -0.10 0.00 0.10 0.20 0.30 0.40
Standardized mean difference
b
QinJ_2012 and JieZ_2017 extrmeness compared to overall sex-related microbial KEGG Orthologs
QinJ_2012, JieZ_2017 average
Meta-analysis coefficient
Effect size
0.2 0.1 0.0 −0.1 −0.2 −0.3 −0.4
K01447: N-acetylmuramoyl-L-alanine amidase
K02022: NO_NAME
K09819: manganese/iron transport system permease protein
K00333: NADH-quinone oxidoreductase subunit D
K09007: GTP cyclohydrolase I
K11784: cyclic dehypoxanthinyl futalosine synthase
K21977: NO_NAME
K03775: FKBP-type peptidyl-prolyl cis-trans isomerase SlyD
K16211: NO_NAME
K00254: dihydroorotate dehydrogenase
K19310: bacitracin transport system permease protein
K09516: all-trans-retinol 13,14-reductase
K01787: N-acylglucosamine 2-epimerase
K00340: NADH-quinone oxidoreductase subunit K
K08307: membrane-bound lytic murein transglycosylase D
K03781: catalase
K16153: phosphorylase / glycogen(starch) synthase
K01130: arylsulfatase
K00060: threonine 3-dehydrogenase
K03389: heterodisulfide reductase subunit B
K10201: N-acetylglucosamine transport system permease protein
K07051: NO_NAME
K19355: mannan endo-1,4-beta-mannosidase
K06295: spore germination protein KA
K00627: pyruvate dehydr. E2 component (dihydrolipoamide acetyltr.)
K01667: tryptophanase
K13652: NO_NAME
K00281: glycine dehydrogenase
K19002: 1,2-diacylglycerol 3-alpha-glucosyltransferase
K20265: glutamate:GABA antiporter
K21571: NO_NAME
K16881: mannose-1-phosphate guanylyltr. / phosphomannomutase
K00970: poly(A) polymerase
K11927: ATP-dependent RNA helicase RhlE
K01596: phosphoenolpyruvate carboxykinase (GTP)
K01991: polysaccharide export outer membrane protein
K06989: aspartate dehydrogenase
K01590: histidine decarboxylase
K18234: NO_NAME
K18011: beta-lysine 5,6-aminomutase beta subunit
K17992: NO_NAME
K03851: taurine-pyruvate aminotransferase
K08717: urea transporter
K12339: cysteine synthase B
K06167: phosphoribosyl 1,2-cyclic phosphate phosphodiesterase
K18866: D-alanyl-D-alanine dipeptidase/carboxypeptidase
K07405: alpha-amylase
K12343: 3-oxo-5-alpha-steroid 4-dehydrogenase 1
K07402: xanthine dehydrogenase accessory factor
K19221: cob(I)alamin adenosyltransferase
K00826: branched-chain amino acid aminotransferase
K03639: cyclic pyranopterin phosphate synthase
K03412: two-component system, chemotaxis family, response regulator CheB
K02760: PTS system, cellobiose-specific IIB component
K01751: diaminopropionate ammonia-lyase
K01854: UDP-galactopyranose mutase
K05942: NO_NAME
K01989: putative ABC transport system substrate-binding protein
K01232: maltose-6'-phosphate glucosidase
K03833: selenocysteine-specific elongation factor

Supplementary Figure 3. Meta-analysis of 5,505 individuals metagenomes from 18 datasets (3,288 females, 2,216 males) reveals sex-associated microbial differences in the KEGG Orthologs potential of the healthy, adult, stool microbiome. **a)** The 30 microbial species and genera with highest Standardized Mean Differences meta-analysis coefficient ($Q < 0.01$) of male vs. female samples. Effect-sizes are computed as Standardized Mean Differences from a linear model controlling for age, BMI, and sequencing depth on centered log-ratio transformed species relative abundances. Yellow: significant effect-size ($Q < 0.2$). Light-blue: non significant effect-size. Black diamonds: Standardized Mean Differences between males and females synthesized by a meta-analysis. Yellow horizontal lines show 95% confidence intervals of the meta-analysis effect size. **b)** Two outlier studies (QinJ_2012, JieZ_2017) from the Random Forest analysis of **Figure 1c** show average stronger associations between sex and KEGG Orthologs microbial carriages compared to the overall meta-analysis, when considering the 60-strongest KEGG associations.

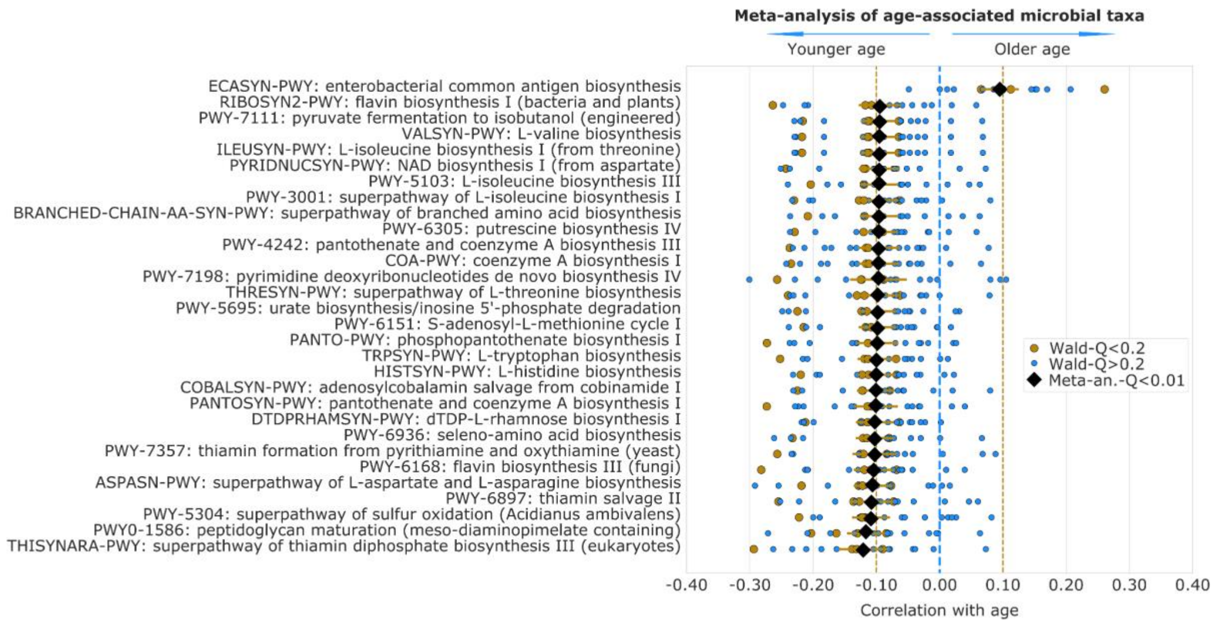

Supplementary Figure 4. Meta-analysis of correlation of age-related (N=4,723) Metacyc pathways microbiome potential - 30 Metacyc pathways having the highest meta-analysis correlation coefficient with participant age (Q < 0.01) with a prevalence of at least 1% in the cohort of adult control participants. Partial correlations are calculated by a linear model controlling for sex, BMI, and sequencing depth using centered log-ratio transformed pathway PRKs. Yellow circles: individually significant effect sizes (Q < 0.2). Light-blue circles: not individually significant. Black diamonds: meta-analysis correlation coefficient computed as a random-effect meta-analysis on Fisher-Z transformed partial correlation. Meta-analysis coefficients and confidence intervals are then reverted back by inverse Fisher-Z transformation. Yellow horizontal lines represent 95% confidence intervals of the meta-analysis correlation.

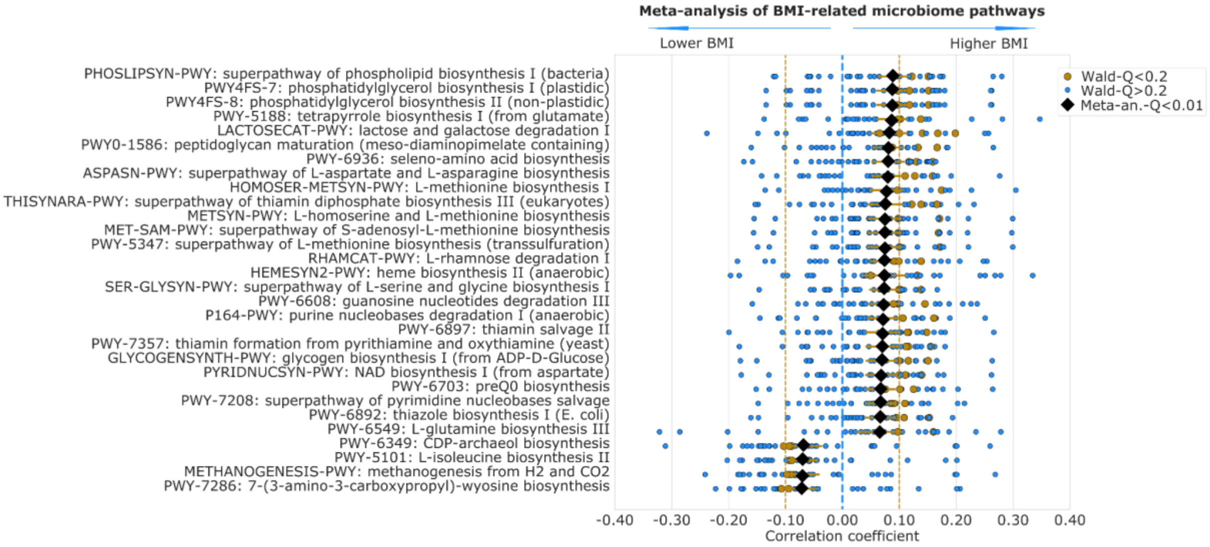

Supplementary Figure 5. Meta-analysis of correlation of BMI-related (N=6,361) Metacyc pathways microbiome potential - a) 30 Metacyc pathways having the highest meta-analysis correlation coefficient with participant BMI (Q < 0.01) with a prevalence of at least 1% in the cohort of adult control participants. Partial correlations are calculated by a linear model controlling for sex, age, and sequencing depth using centered log-ratio transformed pathway RPKs. Yellow circles: individually significant effect sizes (Q < 0.2). Light-blue circles: not individually significant. Black diamonds: meta-analysis correlation coefficient computed as a random-effect meta-analysis on Fisher-Z transformed partial correlation. Meta-analysis coefficients and confidence intervals are then reverted back by inverse Fisher-Z transformation. Yellow horizontal lines represent 95% confidence intervals of the meta-analysis correlation.

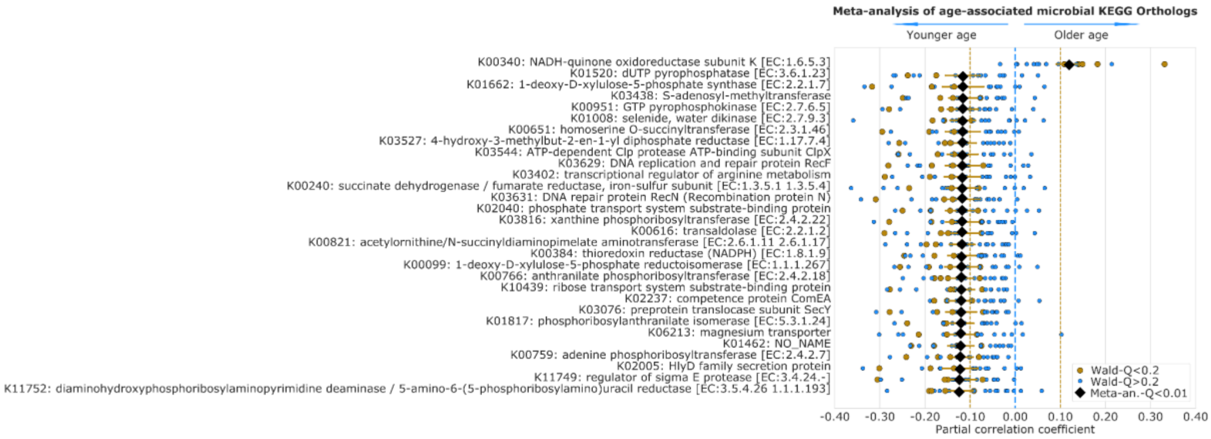

Supplementary Figure 6. Meta-analysis of correlation of age-related (N=4,723) KEGG Orthologs microbiome potential - 30 KEGG Orthologs having the highest meta-analysis correlation coefficient with participant age (Q < 0.01) with a prevalence of at least 1% in the cohort of adult control participants. Partial correlations are calculated by a linear model controlling for sex, BMI, and sequencing depth using centered log-ratio transformed pathway PRKs. Yellow circles: individually significant effect sizes (Q < 0.2). Light-blue circles: not individually significant. Black diamonds: meta-analysis correlation coefficient computed as a random-effect meta-analysis on Fisher-Z transformed partial correlation. Meta-analysis coefficients and confidence intervals are then reverted back by inverse Fisher-Z transformation. Yellow horizontal lines represent 95% confidence intervals of the meta-analysis correlation.

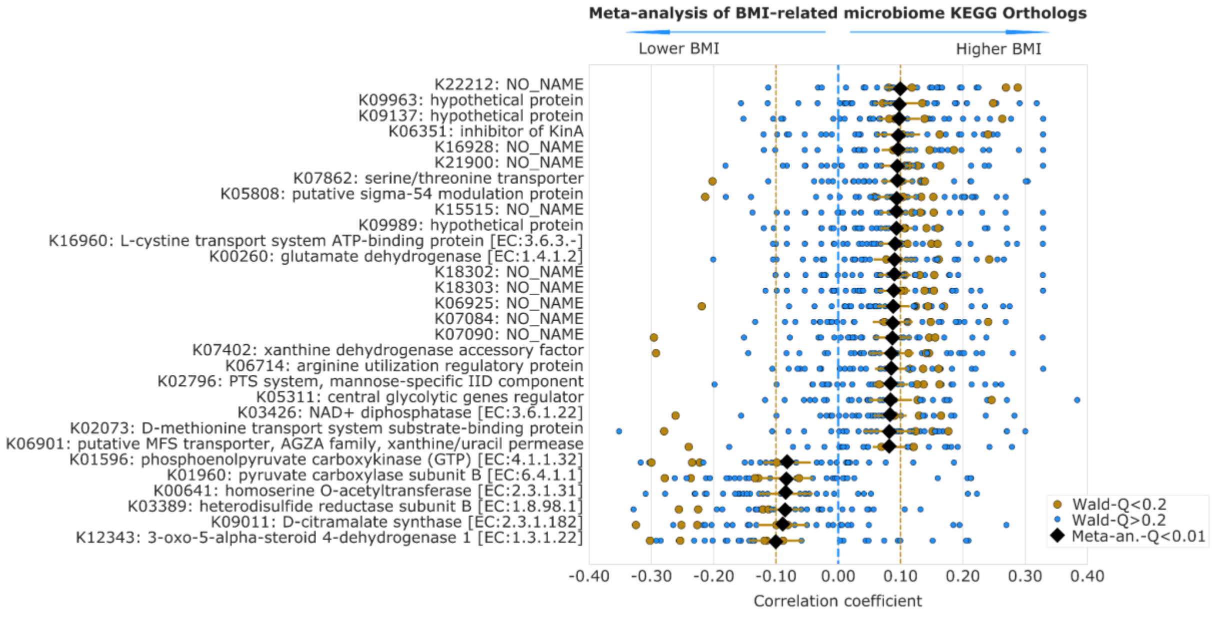

Supplementary Figure 7. Meta-analysis of correlation of BMI-related (N=6,361) KEGG Orthologs microbiome potential - a) 30 KEGG Orthologs having the highest meta-analysis correlation coefficient with participant BMI ($Q < 0.01$) with a prevalence of at least 1% in the cohort of adult control participants. Partial correlations are calculated by a linear model controlling for sex, age, and sequencing depth using centered log-ratio transformed pathway RPKs. Yellow circles: individually significant effect sizes ($Q < 0.2$). Light-blue circles: not individually significant. Black diamonds: meta-analysis correlation coefficient computed as a random-effect meta-analysis on Fisher-Z transformed partial correlation. Meta-analysis coefficients and confidence intervals are then reverted back by inverse Fisher-Z transformation. Yellow horizontal lines represent 95% confidence intervals of the meta-analysis correlation.

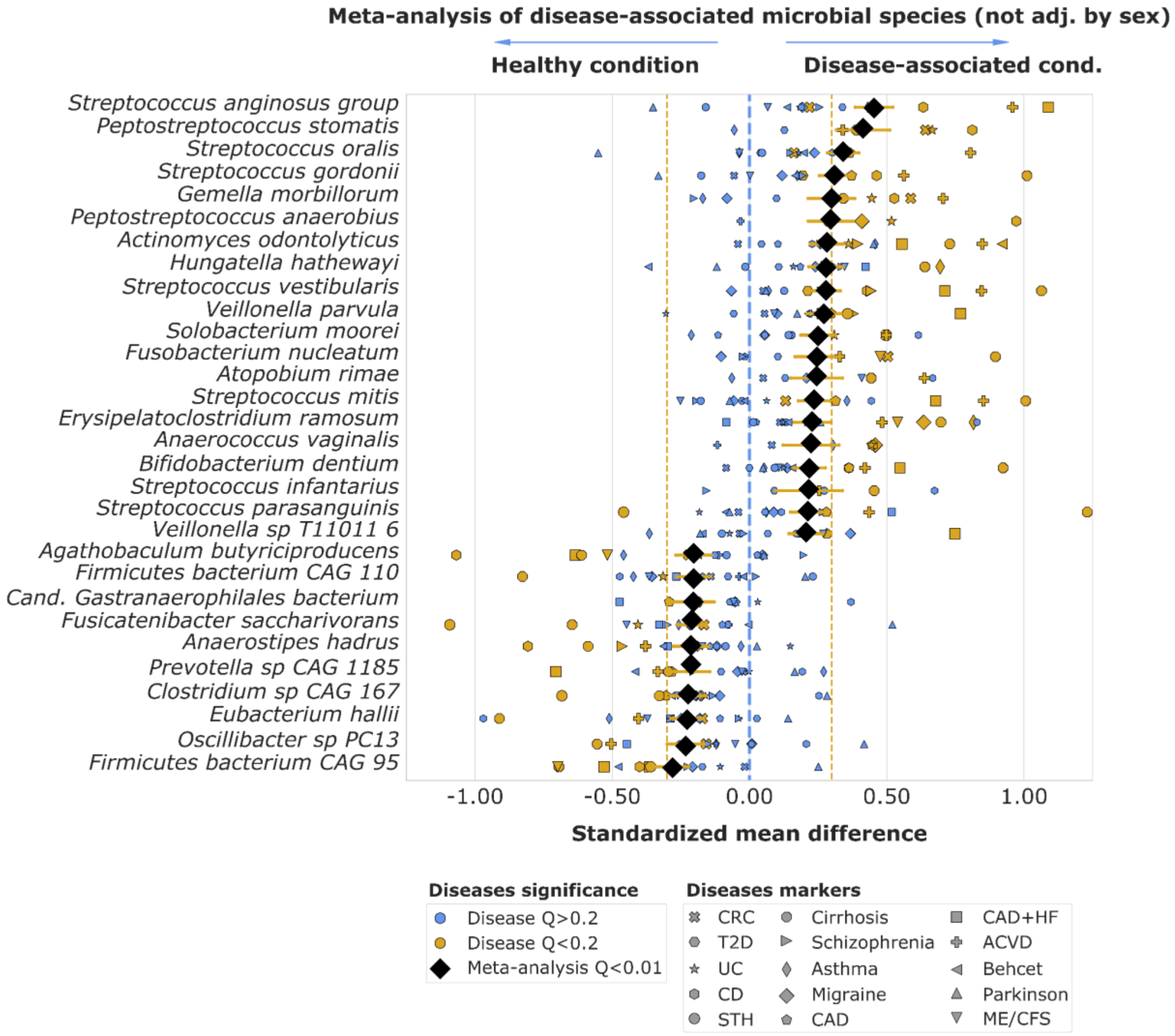

Supplementary Figure 8. Meta-analysis of 15 diseases and 30 cohorts reveals microbial markers of disease or health in 2,346 matched controls and 2,300 diseased patients slightly different with respect to the same analysis performed adjusting also by sex - a) the 30 microbial species with highest meta-analysis coefficient and (Q < 0.01) of disease-associated vs. control samples, with a prevalence of at least 1% in the cohort. Effect-sizes are computed as Standardized Mean Differences from a linear model controlling for age, BMI, and sequencing depth, and usage of antibiotics in the type-2 diabetes datasets, on centered log-ratio transformed species relative-abundances. Acronyms are for: colorectal cancer (CRC), Crohn's disease (CD), ulcerative colitis (UC), type-2 diabetes (T2D), atherosclerotic cardiovascular disease (ACVD), Behcet disease (BD), soil-transmitted helminths (STH), myalgic encephalomyelitis or chronic fatigue syndrome (ME/CFS), coronary artery disease (CAD), coronary artery disease with heart failure (CAD+HF), Parkinson's disease (PD). Effect-sizes of CRC, CD, UC, & T2D are synthesized in a meta-analysis prior to be include in the second meta-analysis, so that these more frequently studied diseases do not dominate the results over diseases for which a single dataset is available (ACVD, asthma, migraine, STH, cirrhosis, ME/CFS, schizophrenia, BD, CAD, CAD+HF, PD). Yellow shape: individually significant effect (Q<0.2). Light-blue shape: non individually significant effect. Black diamonds: Standardized Mean Differences between all cases and controls synthesized by meta-analysis. Yellow horizontal lines show 95% confidence intervals of the synthesized effect size.

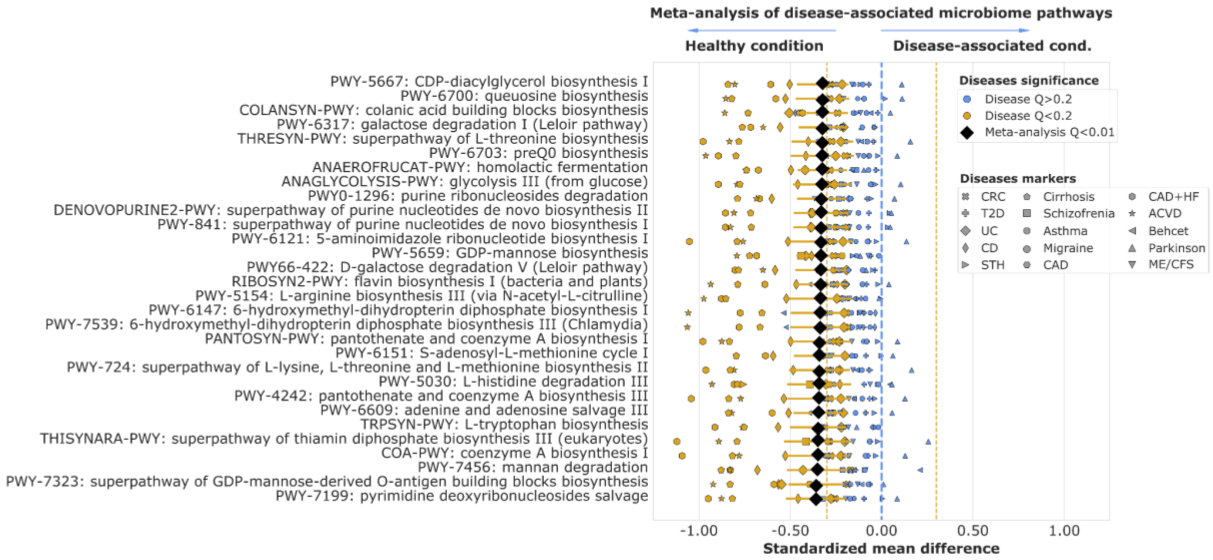

Supplementary Figure 9. Meta-analysis of 15 diseases and 30 cohorts reveals hallmarks of disease in the microbiome Metacyc pathway potential of 2,346 matched controls and 2,300 diseased patients - a) the 30 Metacyc pathways with highest meta-analysis coefficient and Q < 0.01 of disease-associated vs. control samples, with a prevalence of at least 1% in the cohort. Effect-sizes are computed as Standardized Mean Differences from a linear model controlling for sex, age, BMI, and sequencing depth, and usage of antibiotics in the type-2 diabetes datasets, on centered log-ratio transformed pathway RPKs. Acronyms are for: colorectal cancer (CRC), Crohn's disease (CD), ulcerative colitis (UC), type-2 diabetes (T2D), atherosclerotic cardiovascular disease (ACVD), Behcet disease (BD), soil-transmitted helminths (STH), myalgic encephalomyelitis or chronic fatigue syndrome (ME/CFS), coronary artery disease (CAD), coronary artery disease with heart failure (CAD+HF), Parkinson's disease (PD). Effect-sizes of CRC, CD, UC, & T2D are synthesized in a meta-analysis prior to be include in the second meta-analysis, so that these more frequently studied diseases do not dominate the results over diseases for which a single dataset is available (ACVD, asthma, migraine, STH, cirrhosis, ME/CFS, schizophrenia, BD, CAD, CAD+HF, PD). Yellow shape: individually significant effect (Q<0.2). Light-blue shape: non individually significant effect. Black diamonds: Standardized Mean Differences between all cases and controls synthesized by meta-analysis. Yellow horizontal lines show 95% confidence intervals of the synthesized effect size.

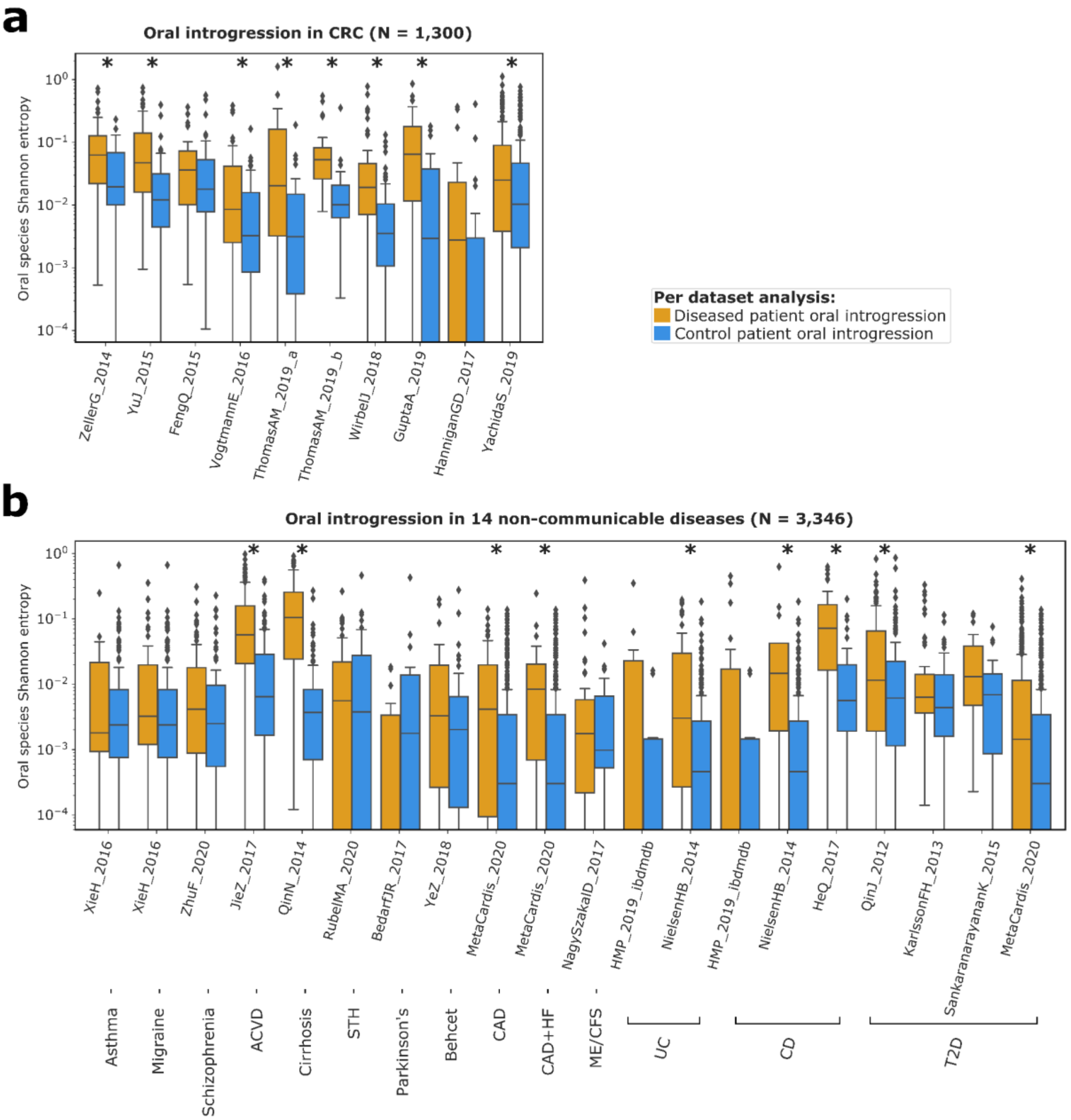

Supplementary Figure 10. Shannon entropy of typically oral taxa in the human gut microbiome is a potential indicator of disease - a) log-10 distributions of Shannon entropy of oral-cavity typical microbial species (defined using 1% of the oral samples as a threshold) in 10 cohorts of CRC patients (orange) and related controls (blue). Asterisks mark the Wilcoxon signed-rank test Q < 0.1. **b)** boxplots showing the log-10 distributions of oral-cavity typical microbial species Shannon entropy (defined using 1% of the oral samples as a threshold) in 14 diseases (20 cohorts), divided by disease (orange) and controls (blue). Asterisks mark the Wilcoxon signed-rank test Q<0.1.

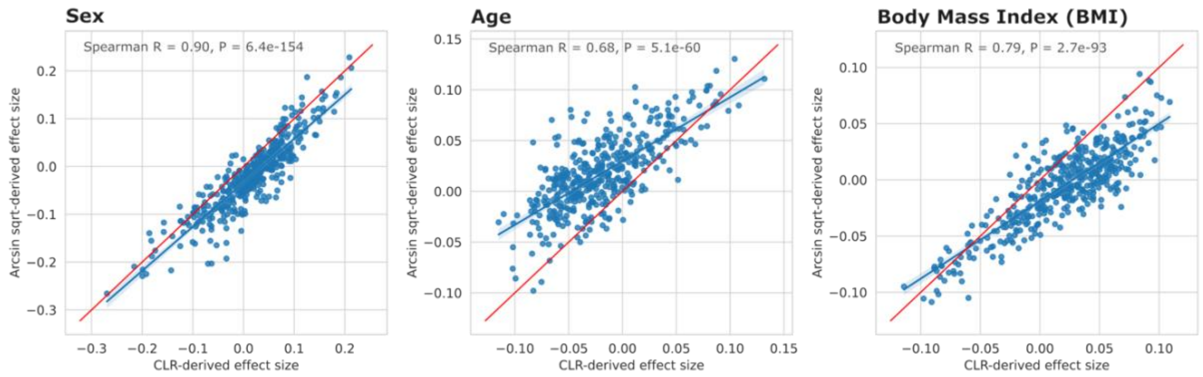

Supplementary Figure 11 - Correlation plots between meta-analysis coefficients of centred log-ratio (CLR) and arcsine square root-transformed species abundances in relation to Sex, Age, and Body Mass Index (BMI). X-axes show coefficients obtained transforming species relative abundances using the CLR, a 1e-5 zero imputation and a filter on prevalence at 5%; the CLR transformation corrects data compositionality. Y-axes show coefficients obtained with the arcsine square root-transformed species relative abundances; the arcsin square root transformation is variance-stabilizing and assumes binomial probability of species non-negativity. The blue line is a correlation trendline, the red line is the diagonal.
