# Supplementary material for: Meta-analysis of 22,710 human microbiome metagenomes defines an oral-to-gut microbial enrichment score and associations with host health and disease
Source: Nat Commun. 2025 Dec 23;17:196. doi: 10.1038/s41467-025-66888-1 (PMC12780261; doi:10.1038/s41467-025-66888-1)
Supplement: Supplementary file 2 — Description of Additional Supplementary Files [file 41467_2025_66888_MOESM2_ESM.docx]

Supplementary Data 1: curatedMetagenomicData 3 datasets included in the analysis. HeQ_2017 is not yet available via the R package.

Supplementary Data 2. Meta-analysis of sex-related microbial species/genera, pathways & KEGGs in 5,505 healthy adults (2,217 males and 3,288 females). Effect sizes are calculated as SMDs from a linear model controlling for age, BMI, and sequencing depth, applied to centered log-ratio transformed species relative abundances. Q-values were calculated from P-values with the Benjamini-Hochberg false discovery rate.

Supplementary Data 3. Meta-analysis of age-related microbial species/genera, pathways & KEGGs in 4,723 healthy adults. Effect sizes are calculated as partial correlations calculated by a linear model controlling for sex, BMI, and sequencing depth using centered log-ratio transformed species and genera relative abundances. Q-values were calculated from P-values with the Benjamini-Hochberg false discovery rate.

Supplementary Data 4. Meta-analysis of BMI-related microbial species/genera, pathways & KEGGs in 6,361 healthy adults. Effect sizes are calculated as partial correlations calculated by a linear model controlling for sex, BMI, and sequencing depth using centered log-ratio transformed species and genera relative abundances. Q-values were calculated from P-values with the Benjamini-Hochberg false discovery rate.

Supplementary Data 5. Meta-analysis of health- & disease-related microbial species & pathways in 4,646 adults (15 diseases, 2,300 cases, 2,346 controls). Effect sizes are calculated as SMDs from a linear model controlling for age, BMI, and sequencing depth, applied to centered log-ratio transformed species relative abundances. Q-values were calculated from P-values with the Benjamini-Hochberg false discovery rate.

Supplementary Data 6. Meta-analysis of Oral Enrichment Scores on 15 different diseases. Scores were calculated at different mean thresholds of prevalence per dataset

Supplementary Data 7. Full taxonomy of the oral species identified with different mean prevalence across dataset cutoffs in oral samples of curatedMetagenomicDatat 3. These can be used to calculate the Oral Enrichment Score.

Supplementary Data 8. Statistics related to panels B to D in figure 5 of the main text
